# Soil-Food-Environment-Health Nexus for Sustainable Development

**DOI:** 10.34133/2021/9804807

**Published:** 2021-04-29

**Authors:** Baojing Gu, Deli Chen, Yi Yang, Peter Vitousek, Yong-Guan Zhu

**Affiliations:** ^1^College of Environmental and Resource Sciences, Zhejiang University, Hangzhou 310058, China; ^2^Zhejiang Provincial Key Laboratory of Agricultural Resources and Environment, Zhejiang University, Hangzhou 310058, China; ^3^School of Agriculture and Food, The University of Melbourne, Melbourne, VIC 3010, Australia; ^4^Key Laboratory of the Three Gorges Reservoir Region's Eco-Environment, Ministry of Education, Chongqing University, Chongqing 400045, China; ^5^Department of Biology, Stanford University, Stanford, CA 94305, USA; ^6^Key Lab of Urban Environment and Health, Institute of Urban Environment, Chinese Academy of Sciences, Xiamen, Fujian 361021, China; ^7^Research Center for Eco-Environmental Sciences, Chinese Academy of Sciences, Beijing 100085, China

## Abstract

Changes in soil properties and processes can influence food and environmental quality, thus, affecting human health and welfare through biogeochemical cascades among soil, food, environment, and human health. However, because many soil properties change much more slowly than do management practices and pollution to soil, the legacy of past influences on soil can have long-term effects on both human health and sustainability. It is essential and urgent to manage soils for health and sustainability through building the soil-food-environment-health nexus.

## 1. Introduction

**Figure 1:**
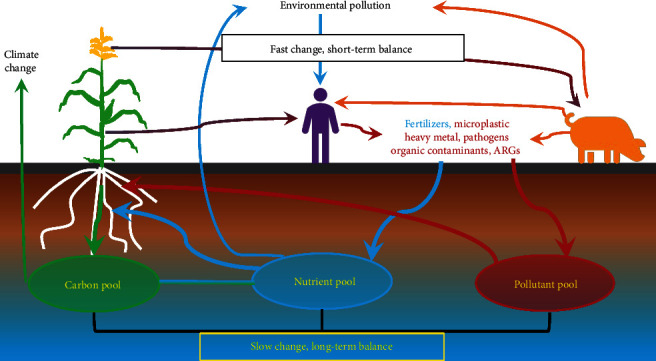
Diagram of soil-food-environment-human nexus. The mismatch of fast changes derived from human activities and slow changes derived from soil processes require the protection of soils urgently. Soil pollution can cascade through the food chain and environment to humans and damage human health ultimately. ARGs refer to antibiotic resistance genes.

Soil is a renewable resource essential for food supply. Soil is also an important sink for atmospheric carbon dioxide (CO_2_) that can contribute to achieving global carbon neutrality [1]. Although the importance of soil is well known, its intricate links to human health and environmental sustainability are poorly appreciated due to a shortage of knowledge on biogeochemical cascades among soil, food, environment, and human health. Most importantly, soil properties can change much more slowly than do management practices, pollution, and their direct effects on human and ecosystem health (Figure 1), thereby representing a legacy that imposes long-term constraints on our ability to manage those effects efficiently. Understanding both slow and fast changes and their mutual linkages is essential not only for food security but also for other global sustainable development goals.

## 2. Nutrients and Climate

Soil nutrients are essential to plant growth. However, pools of these soil nutrients change slowly compared to nutrient input to soil (Figure 1). Either a surplus or a deficiency of soil nutrients can cause long-lasting and difficult-to-reverse changes in soils. Soil nutrient surplus (typically resulting from the accumulation of excessive application of chemical fertilizers) causes air and water pollution, which further cascade through biogeochemical cycles and damage human health and ecosystem sustainability—now and in the future [2]. Excessive use of nitrogen (N) fertilizers led to soil N surplus several decades ago in China, the US, and Western Europe; however, consequent nitrate leaching from maize fields is still an important source of water pollution nowadays [3]. Overuse of N fertilizers can also acidify the soil and thereby causes a series of changes in soil chemical (e.g., loss of cations) and biological properties, which are slow compared to changes of fertilizer input, difficult, and expensive to reverse. The overuse of ammonium fertilizers in China has led to the 1-unit decline of soil pH in many Chinese croplands, damaging soil health and threatening long-term food security [4].

Soil nutrient depletion (typically resulting from harvests that cumulatively remove nutrients in excess of replacement) is commonly found across many countries and regions, including Southern Australia, Northeastern China, and much of Africa. Insufficient and/or imbalanced supply of soil nutrients reduces soil fertility and soil organic matter, and so increases the erodibility of soils [5]. Changes in soils caused by nutrient depletion are also slow compared to soil nutrient input and difficult to recover from, carry the risk of desertification, and reduce soils' resilience to climate change. The low fertilizer application rate in South Australia has led to continuing decline of soil organic matter. Changes in soil nutrients also alter soil microbial communities in ways that could constrain crucial soil functions and threaten food production and safety [6]. Furthermore, the excessive or deficient contents of other elements such as selenium can reduce the long-term nutritional quality of agricultural produces in ways that ultimately impact human health. Yield-oriented crop breeding and soil management can lead to reductions in micronutrient density in food despite more calories being produced, and the enrichment of atmospheric CO_2_ concentrations can further exacerbate such a micronutrient deficiency [7].

Although changes in soil nutrients alter soil carbon pools [8], the slow change of those pools can make it cost-ineffective to manage the soil carbon sink for climate mitigation. Besides, increasing soil carbon often requires N input, and the CO_2_ emissions from producing N fertilizers and nitrous oxide (N_2_O) emission from fertilizer application may offset the amount of soil carbon sequestered. Once soil carbon starts to decline, it is difficult to reverse the loss. Land use changes, especially deforestation for crop production, drive rapid soil carbon loss that contributes to climate change [1]. Management practices that restore carbon storage in agricultural soils through conservation tillage in degraded sites can remove atmospheric CO_2_ and thus mitigate climate change. However, the processes underlying increasing carbon storage in soils typically are on a timescale spanning decades, so it is better to avoid soil degradation in the first place.

These soil legacy effects on nutrients can be further aggravated by long-distance trade. About 15-20% of global grains are traded internationally and consumed by humans or livestock in importing countries where a large amount of excreta is ultimately produced. The nutrients in this excreta cannot be returned to their original soils, decoupling links spatially between soils and nutrients and leading to soil nutrient depletion in exporting countries and surplus in importing countries [9]. Brazil produces one-third of global soybeans, the majority of which are exported to other countries for livestock. Forests and savannas are converted to cultivate soybeans for export. Consequently, soil fertility is declining in Brazil because exported nutrients cannot be returned to agricultural fields. In contrast, Ireland imports a large amount of feed for livestock, which produces excessive manure that is then applied to cropland soils, leading to nitrate runoff and ammonia emissions that degrade local water and air quality. Such long-distance trade breaks the linkage of nutrient cycles within cropland-livestock-human systems and drives persistent soil, environmental, and human health degradation.

Driven by rapidly growing international trade as well as by *in situ* agricultural practices, soil nutrients are changed dramatically. About 70% of Australian wheat is exported to other countries, leaving no chance for closing the loop of nutrient cycles within Australia. The changes derived from trade can also occur on regional scales within a country. The black soil in Northeast China is mainly used for corn, soybean, and high-quality rice production. As an important gain production base, this region has exported a large part of its grain production to other regions within China. These soils thus face the risk of soil degradation and erosion due to a shortage of nutrient recycling and loss of soil carbon. The “Protecting Black Soil Plan” has been proposed to save the soil for sustainable agriculture in China. Carefully designed coupling between soils and livestock and humans can recycle nutrients, reduce environmental pollution, and maintain a sustainable soil-nutrient balance for the long term, even given that over half of global grains are used for animal feed [10]. In the context of fast-growing global trade, increasing nutrient recycling on local scales can save not only nutrients for soils but also the transportation cost of feed for the livestock sector.

## 3. Pollutants

Other than nutrients in soil, pollutants such as heavy metals, plastics, organic contaminants (pesticides, persistent organic pollutants, and emerging organic contaminants), and pathogens can also accumulate irreversibly or very slowly reversibly in soils and pose a significant risk to human health on longer time scales. Heavy metals and organic contaminants are usually toxic to humans, and once taken up, they are difficult to remove and can accumulate in human tissues, finally leading to health issues [11]. Removal of these contaminants in the soil is also slow and difficult, and the impact on human health is chronic. Therefore, the cascading effect of metals and organic contaminants pollution must be recognized well in advance; otherwise, it would be too late when the impact is visible. Rice plants can absorb cadmium and bioaccumulate it in grains and straws, which then cascades through food chains to livestock and human. The continued accumulation of cadmium in humans can damage the kidneys and bones. The cadmium content of rice in Japan's Shintsugawa and Tigawa Basins used to be as high as 1.06 mg/kg, leading to a syndrome of symptoms called “Itai-Itai Disease.” Besides cadmium, nickel, mercury, arsenic, and lead are also commonly found in polluted soils. Globally, there are over 500 million hectares of soil suffering from heavy metal pollution [11]. Complete removal of these contaminants is difficult on field scales once polluted, and amendments are widely used to reduce the uptake of heavy metals by plants. However, the risk is still there once the soil condition has changed or the effects of amendments become weak, emphasizing the importance of protecting soils rather than seeking to manage the consequences of their degradation.

Similar issues can be found in microplastics in soil that are derived from the misuse of plastic film mulch and other plastic products. To increase crop yields, plastic mulch is widely used, mainly in dryland regions to maintain soil moisture and in cold regions to increase soil temperature [12]. However, the low recovery of plastic mulch results in the accumulation of a large amount of plastic film residues in soils that alter their physicochemical properties, threatening plant growth in the long term. Meanwhile, these small pieces of plastic and their additives such as phthalates in soil can be taken up by plants and transferred along the food chain to consumers, including humans [12]. These microplastics have been detected in the excreta of humans in many countries. Furthermore, the chemicals derived from microplastic exhibit endocrine-disrupting, mutagenic, and carcinogenic properties. The biodegradation rate of plastics in soil can range from years to decades, so these negative impacts can also last for decades and even longer in soils.

Soil pathogens are common in soils with continuous cropping. Mismanagement of cropping boosts the growth of soil pathogens that are difficult to control or require a large amount of investment [13]. For instance, the intensive cultivation of ginseng or vegetables can severely damage soil health in the absence of soil restoration. Some of these pathogens can infect animals and/or humans directly from soils or through food chains. Also, antibiotic resistance genes (ARGs) are commonly found in soils with heavy use of manure derived from animal agriculture in which antibiotics are used in intensive feedlots [14]. These soil organisms can persist for many years, damaging food safety and threatening human health.

## 4. The Nexus

From soil to human and vice versa is an intricate linkage, within which managing the cascade between soil and human on both micro- and macroscales is critical. Consideration of the soil-food-environment-health nexus is required to address food security and Sustainable Development Goals [15]. The negative impacts outlined above are slow compared to those in soil management practices; therefore, maintaining existing soil health and restoring that of degraded soils will be the key to managing the soil-food-environment-health nexus. Without managing this nexus, soil health will be at long-term risk, ultimately damaging food-environment-health. The long-term nature of soil pollution and degradation, and the rate, expense, and difficulty of their remediation require a soil-food-environment-health nexus approach to maintain soil health.

Based on the nexus approach, our understanding of soils should focus on the slow soil processes if we are to benefit from managing the fast processes. This requires more comprehensive assessment of our activities such as manure recycling and plastic film use to quantify not only short-term outcomes but also long-term impacts on slowly changing soil properties. In addition to crop yields, we should consider food safety, ecosystem, and human health using the nexus approach. We suggest that multiple goals of soil health should be managed simultaneously, and more preventive measures should be implemented before soils become degraded. Urgent actions are required to manage soils from local to global scales with involving multiple stakeholders from governments, farmers, publics, and scientists. Legislation to protect soils by governments should take the entire soil-food-environment-health nexus into consideration in the context of global trade. Such legislation should focus on long-term processes that support farmers in maintaining soil health on at least decadal scale with multiple goals and financial supports from governments and public sectors. Optimizing public behaviors would substantially reduce the pressure driving soil degradation such as dietary change. More soil-friendly behavior can help to achieve the nexus. Nevertheless, before harnessing these intricate interconnections across the nexus, more comprehensive understanding of the nexus should underpin the launching of any science-based management.

Further research should develop quantitative indicators that can help measure the performance of conservation practices across the soil-food-environment-health nexus. Those practices that maximize the long-term and overall benefits of the nexus areas should be promoted on existing cultivated land in accordance with local conditions. Conservation agriculture such as no tillage has both short-term benefits like reducing the use of fertilizers and pesticides, thus, improving water quality, and reducing soil erosion, and long-term benefits like increasing soil carbon storage. Restoration of abandoned lands should also be given priority because such areas are mostly degraded and could remain a source of environmental concern in the long run (e.g., through erosion and water pollution) given the slow changes of soil. Land abandonment has been a common phenomenon worldwide, especially in developed countries, and will continue to occur for various reasons [16]. Greater resources should be devoted to accelerating the soil health of abandoned and polluted lands (e.g., via the restoration of vegetation diversity) so that they can contribute to future food security, environmental sustainability, and human health.

## Data Availability

The data is available from the authors.
